# Heart Failure with Preserved Ejection Fraction in Egypt: An Expert Opinion

**DOI:** 10.5334/gh.1411

**Published:** 2025-03-20

**Authors:** Magdy Abdelhamid, Amr Zaki Salem, Hamza Kabil, Hany Ragy, Hosam Hasan-Ali, Mohamed Elnoamany, Mohamed Elsetiha, Sameh Shaheen

**Affiliations:** 1 Faculty of Medicine, Cairo University, Cairo, Egypt; 2 Faculty of Medicine, Alexandria University, Alexandria, Egypt; 3 Faculty of Medicine, Damietta University, Damietta, Egypt; 4 National Heart Institute, Cairo, Egypt; 5 Faculty of Medicine, Assiut University, Assiut, Egypt; 6 Faculty of Medicine, Menofia University, Menofia, Egypt; 7 Faculty of Medicine, Tanta University, Tanta, Egypt; 8 Faculty of Medicine, Ain Shams University, Cairo, Egypt

**Keywords:** heart failure, heart failure with preserved ejection fraction, Egypt, comorbidities

## Abstract

Heart failure with preserved ejection fraction (HFpEF) is an ongoing challenge for healthcare systems. Major limitations that hinder adequate control of the disease, including an incomplete understanding of its pathophysiology, limited therapy options, and the absence of sufficient information on the management of comorbidities. Diagnosis and management of HFpEF in Egypt lack standardization as they are complicated with multiple comorbidities and limited by the lack of resources and data on epidemiology and patient characteristics. Diagnostic procedures for HFpEF should be implemented through guideline-specified scoring systems, due to the heterogeneity of clinical presentations and the absence of a golden standard for confirming HFpEF. In Egypt, the H_2_FPEF scoring system is more commonly used for establishing HFpEF diagnosis. All HFpEF patients should be treated through multidrug regimens tailored for their state, symptoms, and comorbidities, with sodium-glucose cotransporter-2 (SGLT2) inhibitors as the mainstay of treatment together with either one or a combination of loop diuretic and aldosterone antagonists. This paper provides an integrated review of epidemiology, means of diagnosis, current and novel pharmacological therapy options for HFpEF patients in the light of the recent advances in treatment of HFpEF, discussing means of healthcare delivery and unmet needs, and proposing recommendations for clinical practice and pathways for future research.

## Central Illustration

**Figure:**
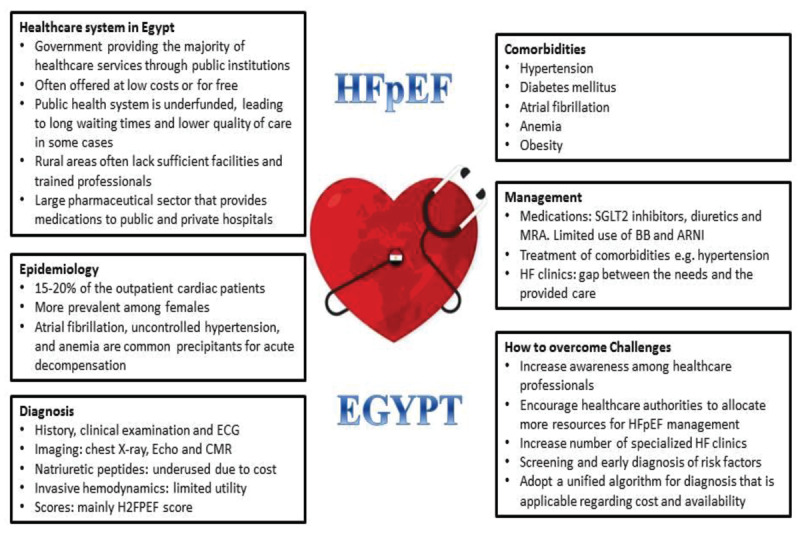


## Introduction

Heart failure (HF) is a complex clinical syndrome with a global prevalence of 1%–3% (1), which drives the need for a better understanding of its nature, diagnosis, categorical classifications, and pharmacological management. For many years, HF was only linked to systolic dysfunction, and reduced left ventricular ejection fraction (LVEF) was essential for HF diagnosis. This remained until HF with normal LVEF was first recognized in clinical practice in the 1980s (2). The cut-off point of 50% EF was used to separate heart failure with reduced ejection fraction (HFrEF) and heart failure with preserved ejection fraction (HFpEF) (3,4,5). Since then, efforts have been directed toward studying HFpEF, establishing means for its diagnosis, and optimizing its clinical management. Despite all these efforts, the optimal level of care for patients is far from reached.

Compared to HFrEF, HFpEF is associated with more comorbidities, including hypertension, chronic kidney disease (CKD), atrial fibrillation (AF), obesity, iron deficiency, and chronic obstructive pulmonary disease (COPD) (3). Both HFrEF and HFpEF show similar hospitalization and all-cause mortality rates (4), but HFpEF is associated with higher rates of non-CV-caused deaths and lower rates of CV-caused deaths compared to HFrEF (4,5,6,7,8).

Moreover, the treatment of HFpEF is an ongoing challenge to healthcare systems due to the very limited therapeutic options. In fact, until very recently, there were no approved or effective therapies that reduce mortality and morbidity, which resulted in empiric and symptom-based management of HFpEF combined with treatments for the comorbidities.

Egypt’s healthcare system is a mix of public and private services, with the government providing the majority of healthcare services through public institutions. The Ministry of Health and Population is the central body responsible for formulating health policies, providing public health services, and ensuring the implementation of health programs across the country. The public healthcare system in Egypt is extensive and includes a network of government-run hospitals, primary healthcare centers, specialized medical institutions, and University Hospitals. These services are generally subsidized and often offered at low costs or for free, especially for citizens in rural areas or those unable to afford private care.

Egypt has a mixed health insurance system. Public health insurance covers certain segments of the population, including government employees and some workers in the formal private sector. The Ministry of Health and Population is working to expand health insurance to cover more citizens through its Universal Health Insurance System (UHIS), which aims to provide coverage for all Egyptians. Private health insurance offers supplementary or primary coverage for individuals who can afford to pay for private insurance plans.

The Egyptian healthcare system has a large pharmaceutical sector that provides medications to both public and private hospitals. The government also regulates drug prices to ensure affordability. Many generic drugs are produced domestically, making them more accessible to the population however the prices of drugs have been increasing in the last few years due to high inflation rate which increases burden over the government and the patients.

Despite its extensive public health services, Egypt faces several challenges in healthcare. First, unequal access to healthcare where rural areas in particular often lack sufficient healthcare facilities and trained professionals. Second, healthcare financing as the public health system is underfunded, leading to long waiting times and lower quality of care in some cases. Third, Egypt’s large and growing population, combined with an increasing burden of chronic diseases like diabetes and hypertension, places additional strain on the healthcare system.

## Objective

The aim of this article is to provide an expert opinion on HFpEF clinical practice in Egypt in light of the recent updates in the diagnosis and management of HFpEF and their applicability to a low-to-middle income country (LMIC).

## Methodology

**Figure 1:**
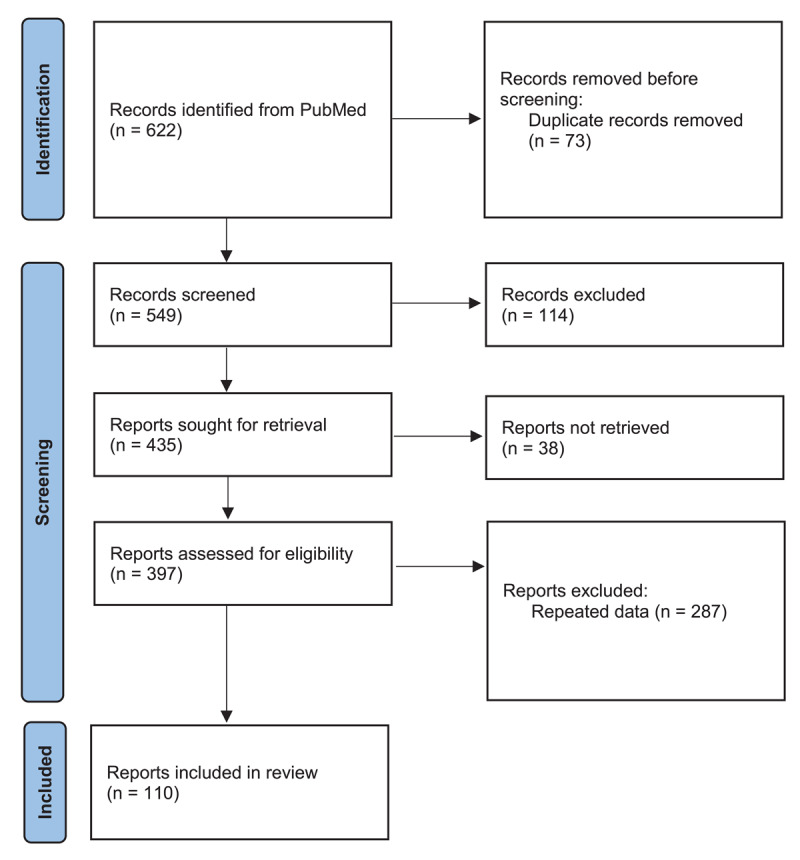
PRISMA systematic review and meta-analysis flow chart.

A panel composed of seven cardiologists with extensive experience in treating HF in the public and private healthcare settings in Egypt met on January 15, 2022. The Preferred Reporting Items for Systematic Reviews and Meta-Analyses (PRISMA) guidelines were followed to conduct the literature search, data extraction, and reporting. Searches were performed on the public database (PubMed) in the last 3 years using the term ‘diagnosis and management of heart failure with preserved ejection fraction’. All papers published from 1 July 2021 to 1 July 2024 were included (Figure 1).

Articles that were in line with the objective of this paper were discussed with the authors’ HFpEF clinical practice insights in the Egyptian healthcare system.

## Discussion

### HFpEF epidemiology in Egypt

Worldwide, HFpEF patients represent 50% of the HF population (9). In Egypt, HF patients represent an average of 15%–20% of the outpatient cardiac patients, and 40%–50% of them are HFpEF patients. Adoption of the western lifestyle, increased rates of obesity, cigarette smoking, and other cardiovascular risk factors (e.g. diabetes mellitus) are contributing factors for significantly higher rates of atrial fibrillation (AF), uncontrolled hypertension, and anemia as precipitants for acute decompensation in HFpEF patients (10).

In agreement with worldwide data, HFpEF is more prevalent among females in Egypt (1,11). In an analysis of data from the Egyptian cohort of the European Society of Cardiology Heart Failure (ESC-HF) Long-term Registry, 29.7% of the females in the study presented with HFpEF compared with 10.6% of males (11). One of the major differences between men and women presenting with HF was obesity. Women admitted with HF were more frequently obese than men. Obesity has been consistently associated with left ventricular hypertrophy and dilatation, which are known precursors of HF. Atrial fibrillation was another frequent feature among women presenting with HF. This may be related to the increased prevalence of hypertension among women (11).

### Clinical diagnosis components

Accurate diagnosis of HFpEF may be tough due to its heterogeneous clinical presentation. Patients may present with little or no symptoms at rest and many of their symptoms may be attributed to old age or non-vascular comorbidities (12). Moreover, there is no single marker that can confirm a diagnosis of HFpEF corresponding to reduced EF as is the case in HFrEF, so a diagnostic road map is essential for a straightforward diagnosis. HFpEF diagnosis is triple-tiered; and evidences of HF, normal EF, and diastolic dysfunction should all contribute to establishing a diagnosis of HFpEF (13).

Accordingly, the first step of diagnosing HFpEF is physical examination and assessing medical history. HFpEF patients typically present with exertional dyspnea, orthopnea, paroxysmal nocturnal dyspnea, congestion, peripheral edema, and chest discomfort, along with a history of AF, diabetes, obesity, or metabolic syndrome (14,15,16). However, patients may present with insensitive or non-specific symptoms that can be explained by many non-cardiac abnormalities (for example, unexplained dyspnea) (15).

Routine laboratory workup recommended for assessing HFpEF includes complete blood count, liver functions, kidney functions, HbA1c, thyroid stimulating hormone, ferritin, serum potassium and sodium, cardiac troponins in case of acute HF and to detect acute coronary syndrome, and D-dimer if pulmonary embolism is suspected (13,14).

A chest X-ray is recommended to investigate other potential causes of dyspnea (e.g. pulmonary disease). It may also provide supportive evidence of HF (e.g. pulmonary congestion or cardiomegaly) (13).

Electrocardiography (ECG) is of a relatively low value in HFpEF diagnosis; however, it is used to detect AF which suggests underlying HFpEF (8). ECG for HFpEF patients could also demonstrate signs of features of LV hypertrophy (LVH) and/or left atrial (LA) enlargement (14). In contrast, echocardiography is essential in the diagnostic work-up for HFpEF. It offers a comprehensive, non-invasive examination of diastolic function and filling pressures through various cardiac parameters, including LVH, mitral valve velocities, mitral annual velocity, E/e’ ratio, LA size, and tricuspid regurgitation (TR) jet velocity (14,16,17). All patients with dyspnea should undergo echocardiographic testing to assess structural abnormalities and determine the EF, to exclude conditions such as HFrEF, valvular disease, pulmonary hypertension, and pericardial effusion. However, the sensitivity of echocardiography is to detect relevant diastolic dysfunction and elevated filling pressures in patients with unexplained dyspnea and a preserved left ventricular ejection fraction is low (35%) (18). In some cases, stress echocardiography may be essential to unmask symptoms that were absent at rest. Using trans-mitral E/e’ and left atrium strain over other more complicated parameters can increase the diagnostic value of echo for detecting HFpEF (19,20). However, clear cut-off points for stress echocardiography parameters in HFpEF are still lacking (21).

Cardiac magnetic resonance (CMR) imaging can be useful in diagnosing HFpEF as it measures LA and LV mass and volumes with the most accuracy. It is mainly used when echocardiography imaging is of suboptimal quality and inconclusive results. It is also useful for assessing myocardial ischemia and/or fibrosis and excluding other conditions that may be confused for HFpEF e.g. hypertrophic cardiomyopathy, amyloidosis, etc. (14,22). Due to its high cost in Egypt, it is not readily available in treatment centers nor is it widely employed by cardiologists.

On the other hand, and despite being a gold standard in diagnosing HF (23), natriuretic peptides (BNP and NT-proBNP) levels testing is highly underused in Egypt due to their high cost. Efforts are now directed toward incorporating natriuretic peptides (NPs) in the HF diagnostic guidelines in the Egyptian healthcare centers, especially that higher NPs levels often indicate co-existing conditions such as AF and COPD (24). Additionally, it should be noted that the concentration of NPs varies with age, sex, kidney function, and obesity; and can even be normal in some HFpEF patients (8,15,25).

Invasive hemodynamics tests can be useful in the clinical diagnosis of HFpEF. However, because resting tests may risk underdiagnosis of patients at the early stages of the disease, invasive exercise hemodynamics testing is recommended but costly which may be problematic in an LMIC. The test is done through standard right heart catheterization or an LVEDP-measurement if a coronary angiogram is considered anyway. Exercise right heart catheterization using a supine cycle ergometry device (which is considered the gold standard for HFpEF in combination with other clinical investigations) is more challenging to organize and possibly not doable in LMIC. The passive leg-raising method during a right heart catheterization is more applicable and can be helpful to diagnose or rule out HFpEF which may prevent the necessity to exercise (26,27). Right atrium (RA) pressure, pulmonary artery (PA) pressure, pulmonary capillary wedge pressure (PCWP), and left ventricle at end-diastole pressure (LVEDP) are then measured (27).

### Diagnostic guidelines and scoring systems

Guidelines for HFpEF diagnosis involve the use of scoring systems to overcome the clinical picture variations in HFpEF. There are currently two scoring systems in use.

The European Society of Cardiology HFA-PEFF algorithm is a stepwise approach starting from clinical assessment to more specialized invasive tests composed of four steps. The first step is pre-test assessments (P) during which patients with suspected HFpEF undergo clinical assessment of their signs and symptoms, assessments of any comorbidities and identification of risk factors, and undergo basic non-invasive cardiac tests such as standard echocardiography, ECG, NPs and 6-min walk test (6MWT), in order to recognize or exclude possible causes for their symptoms. Patients whose results suggest possible HFpEF will move on to the second step (E) (diagnostic workup; comprehensive echocardiographic and NPs score [if the latter was not measured earlier]) and the patient is given a score from 0 to 6. If the patient’s score does not confirm or exclude HFpEF (i.e., from 2 to 4), the patient will undergo the advanced workup step F1 (functional testing due to uncertainty) composed of stress echocardiography and invasive hemodynamics testing. The last step is the etiological workup (F2; final etiology) in which the underlying reason for the diagnosed HFpEF is determined (14,28,29). The drawback of this algorithm is that many patients may be unable to take exercise-based tests or undergo invasive testing (29).

**Figure 2:**
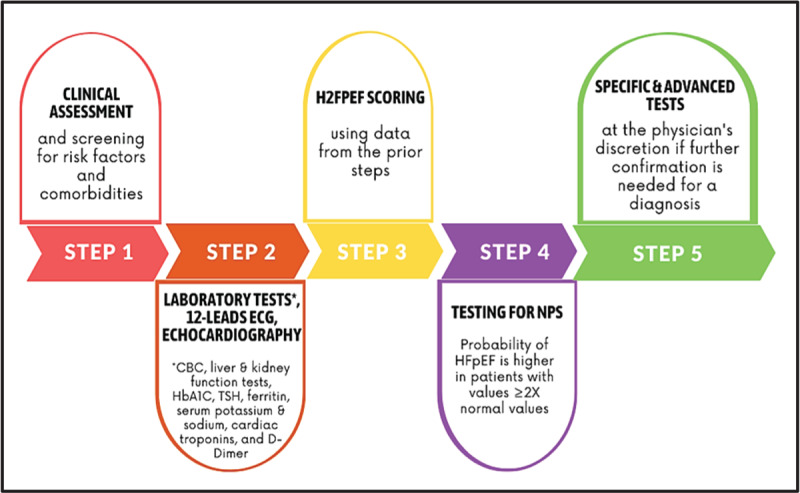
Suggested diagnostic algorithm for HFpEF in Egypt.

The second scoring system is the H_2_FPEF score which consists of 6 variables upon which a final score from 0 to 9 is calculated, with a score above 6 suggesting HFpEF with a probability of 90%. The 6 variables used and abbreviated as H_2_FPEF are a BMI of 30 kg/m^2^ or more (H), the use of two or more antihypertensives (2), atrial fibrillation (F), presence of pulmonary hypertension (P), elderly of age more than 60 years (E), and finally, elevated filling pressure expressed through an E/e’ ratio of more than 9 (F). Each variable is calculated as one point except for AF and BMI, they correspond to three and two points, respectively (30). Both scoring systems are validated with an acceptable diagnostic value and are used widely (31). In Egypt, the H_2_FPEF score is used more commonly as it is more convenient to use and less expensive. Figure 2 presents a suggested diagnostic algorithm for HFpEF in Egypt.

### Quality of life (QOL) assessment questionnaires

Patient perceptions have a great influence on their health outcomes, and patient-reported outcomes (PROs) can give useful insights into disease progression and therapy effectiveness. Accordingly, QOL questionnaires are essential tools in HFpEF management plans, especially in the long absence of therapeutic options (32). Two QoL assessment questionnaires can be used in HFpEF, the Kansas City Cardiomyopathy Questionnaire (KCCQ) and the Minnesota Living with Heart Failure Questionnaire (MLHFQ). Both are validated with accurate and reliable predictive values (33,34). However, in practice, KCCQ use is preferred as it is more in line with other functional assessments like the NYHA classification and the 6MWT (35).

### HFpEF management

For many years, the management of HFpEF aimed exclusively at improving patients’ QoL and functional abilities through managing symptoms and associated comorbidities. Guidelines only recommended loop diuretics to treat congestion and volume overload in the absence of pharmacological interventions proven to reduce morbidity or mortality (13). This was mainly attributed to a gap in understanding the pathophysiological mechanisms of HFpEF.

Empagliflozin, a sodium-glucose co-transporter 2 (SGLT2) inhibitor, was the drug to break this circle in February 2022 when it was approved by the US Food and Drug Administration (FDA) based on its results in the EMPEROR-Preserved study (36,37). The study observed early and consistent reductions in all-cause hospitalizations, mortality, worsening HF events reflected in reducing urgent care outpatient visits and lessening the decline in estimated glomerular filtration rate (eGFR) in HFpEF patients with or without type 2 diabetes (37,38,39). The renoprotective action of empagliflozin also extends to natriuresis, osmotic diuresis, and uric acid reduction (40).

The effect of dapagliflozin, another SGLT2 inhibitor, on morbidity and mortality in HFpEF patients was assessed in DELIVER, a large randomized multicenter study. The use of dapagliflozin in patients with HF with mildly reduced and preserved EF was associated with lower symptom burden, fewer worsening HF events, and less cardiovascular deaths (41). In the 2023 AHA/ACC/HFSA guideline for the management of HF, SGLT2 inhibitors (dapagliflozin and empagliflozin) carry a class IA recommendation in patients with HFpEF to reduce the risk of HF hospitalization or CV death (42).

ACE inhibitors and angiotensin receptor blockers are widely used in HFpEF patients to control the associated hypertension. Although inhibition of the renin-angiotensin-aldosterone system (RAAS) may have positive effects on endothelial damage and in turn cardiac function, treatment with RAAS inhibitors was not proven to reduce mortality or morbidity in HFpEF (43). On this account, the first angiotensin receptor neprilysin inhibitor (ARNI) sacubitril/valsartan was introduced, it combines inhibition of RAAS and neprilysin enzyme which is responsible for the breakdown of natriuretic peptides and other vasoactive peptides (44). Sacubitril/valsartan reduced hospitalizations in HFrEF compared to ACE inhibitors alone (45). However, it showed a nonsignificant reduction in the composite endpoint of total hospitalizations for HF and CV death compared to valsartan in HFpEF in the PRAGAON-HF trial (46). Nevertheless, the FDA expanded its approval of sacubitril/valsartan to patients with guideline-defined HFpEF in early 2021 (47). In Egypt, sacubitril/valsartan is only approved for HF with reduced ejection fraction.

Mineralocorticoid receptor antagonists (MRAs), primarily spironolactone, are also one of the available therapeutic options for HFpEF. Aldosterone antagonists influence diastolic dysfunction, in an effect known as reverse cardiac remodeling, by opposing the endothelial dysfunction, LV hypertrophy, and vascular stiffness effects that aldosterone exerts as a component of RAAS (48,49). Reverse remodeling effect of spironolactone was observed in HFpEF patients; however, these effects were not associated with clinical improvement reflected on patients’ QOL and exercise capacity (49). Moreover, the TOPCAT study reported that treatment with spironolactone showed no significant reduction in either the mortality or the HF hospitalization rates. Nevertheless, the study suggested that certain populations (such as females) may benefit more from MRAs. Accordingly, further research is needed to determine if Egyptian patients’ characteristics are associated with increased benefit (13,50).

The use of beta blockers in HFpEF showed no improvement in all-cause mortality or hospitalizations (51,52), so it should be avoided/down-titrated if possible unless there is a compelling indication such as AF and coronary artery disease (CAD) (53).

**Figure 3:**
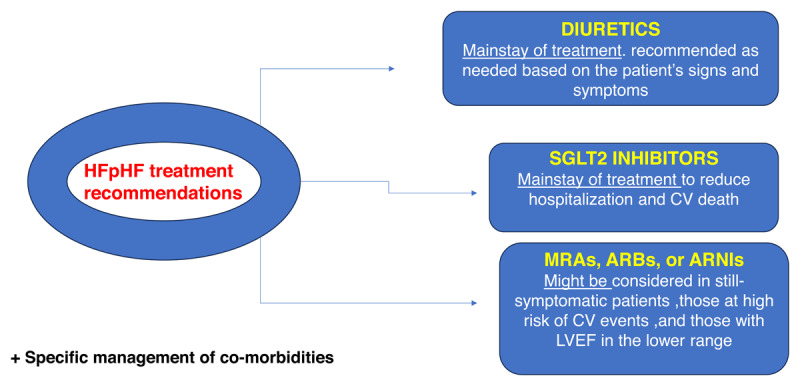
Suggested treatment algorithm for HFpEF in Egypt.

Finally, patients with HFpEF and pacemakers, treatment with a moderately accelerated, personalized pacing rate seems to be safe and improved quality of life, NT-proBNP levels, physical activity, and AF compared with the usual 60 bpm setting (54). Figure 3 shows our suggested algorithm for HFpEF treatment.

### Comorbidities

HFpEF is commonly associated with a wide range of comorbidities, including hypertension, iron deficiency, CAD, CKD, AF, diabetes mellitus, obesity, obstructive sleep apnea, and cerebrovascular disease. These comorbidities should be treated according to the guidelines specified for each of them, as there is not enough data on whether HFpEF affects their course of the disease and in turn their management plans (13).

Recently in the last 5 years, Egypt has developed a National Non-Communicable Diseases Program, which focuses on the early detection, prevention, and management of diseases like hypertension and diabetes mellitus. The program includes conducting regular screenings at both community levels through mobile units in workplaces and public areas and in healthcare settings. Also training healthcare professionals to identify and manage hypertension and diabetes mellitus.

Obesity is one of the strongest risk factors for HFpEF. Obese patients with HFpEF have reduced skeletal muscle mass and increased thigh muscle fat infiltration and intra-abdominal fat mass, all of which are related to their reduced exercise capacity. Caloric restriction (CR) and aerobic exercise training (AT) significantly increased peak exercise oxygen consumption (VO2peak), and their effects were additive. These data supported combined CR+AT as a novel treatment to improve exercise intolerance and QOL in older patients with obese HFpEF (55).

Obstructive sleep apnea (OSA) may be a significant risk factor for HFpEF. The gold standard for diagnosing OSA involves simultaneous monitoring of sleep and breathing, so-called polysomnography. Treatment of OSA in patients with HFpEF with positive airway pressure therapy is associated with reduced risk of the composite outcome of hospitalizations and visits to the emergency room (56). Positive airway pressure therapy is readily available in Egypt and can be easily delivered to patients in collaboration with pulmonologists.

AF ablation improves invasive exercise hemodynamic parameters, exercise capacity, and quality of life in patients with concomitant AF and HFpEF (57); however, due to its high cost, it might not be applicable to offer it to all our patients.

### Heart failure programs and clinics

The HF management system in Egypt needs further adjustments to meet patients’ needs, limit HF prevalence, and lessen the burden on patients, physicians, caregivers, and the Egyptian healthcare system. Establishing new HF-specialized clinics across the country may be the solution to the growing gap between the needs and the care provided. The clinic will be responsible for diagnosing HF, assigning guideline-directed medical therapy, and planning and implementing follow-up plans for patients for as long as they need, in addition to offering a follow-up regimen for patients discharged after hospital admissions. Further research directed at the optimization and development of this HF clinic model is needed to facilitate its incorporation into the Egyptian healthcare system.

### Patient follow-up and treatment adherence

Complexity of treatment plans along with the burden of associated comorbidities is commonly encountered by poor patient compliance. To alleviate this issue, each patient should receive proper education tailored for their needs and level of understanding. In addition, a detailed follow-up plan should be set into consideration to the patient’s preferences and conditions. Moreover, it is important for physicians to discuss psychological aspects and possible barriers to medications’ availability and talk through options for mental and financial support whenever needed.

It is essential to frequently monitor the deterioration of patient’s state and any progress of comorbidities, especially for patients who are unstable, recently diagnosed and undergoing medication up-titration, or recently discharged after a period of hospitalization. Required follow-up measures for HFpEF are not specified by guidelines (13).

### Unmet needs in HFpEF

The diagnosis of HFpEF is still challenging in Egypt owing to the performance variations of diagnostic algorithms in clinical settings and the massive underuse of NPs (13). Moreover, many healthcare workers, especially in the Egyptian settings, lack access to some of the tests needed for both scoring systems, and patients may not afford the needed diagnostic and management procedures (13,58). Additionally, the absence of trained nurses dedicated to HF patients exacerbates those unmet needs. Absence of sufficient HF clinics with dedicated inpatient wards in Egypt leads to outpatient management of patients who should optimally be hospitalized. Lastly, establishing pharmacological drug options for HFpEF is still in its infancy stage, and patients remain in need of more tailored effective treatments for every stage of the disease.

## Conclusion and Future Perspectives

After more than two decades of recognizing HFpEF as a chronic disease, there are still major limitations in our understanding of the best strategies for prevention, diagnosis, and management. More effort is needed to reach a global consensus on HFpEF management in clinical practice. Guidelines for the specific management of HFpEF should be implemented.

We need to increase awareness among our primary care physicians to look for HFpEF among patients presenting with exertional shortness of breath as usually these patients are missed especially if they have essentially normal echocardiography study at rest. More efforts should be directed toward early diagnosis and management of risk factors such as hypertension and diabetes. Awareness campaigns through social media aiming at improving lifestyle, healthy dietary habits, encourage physical exercise, and weight reduction.

The HFpEF clinical practice in Egypt still needs to match global advances in many aspects to improve diagnosis accuracy and introduce more management options.
